# Dissociable Effects of Psychopathic Traits on Executive Functioning: Insights From the Triarchic Model

**DOI:** 10.3389/fpsyg.2018.01713

**Published:** 2018-09-12

**Authors:** Rita Pasion, Ana R. Cruz, Fernando Barbosa

**Affiliations:** Laboratory of Neuropsychophysiology, Faculty of Psychology and Educational Sciences, University of Porto, Porto, Portugal

**Keywords:** antisocial behavior, psychopathy, impulsivity, cognition, executive functioning, personality

## Abstract

The relationship between executive functioning and psychopathy lacks consistent findings. The heterogeneity of the psychopathic personality structure may contribute to the mixed data that emerged from clinical-categorical approaches. Considering the link between antisocial behavior and executive dysfunction from the perspective of the Triarchic Model of Psychopathy, it is suggested that executive impairments in psychopathy are specifically explained by meanness and disinhibition traits, reflecting externalizing vulnerability. In turn, boldness is conceptualized as an adaptive trait. The current study assessed updating (N-back), inhibition (Stroop), and shifting (Trail Making Test) in a forensic (*n* = 56) and non-forensic sample (*n* = 48) that completed the Triarchic Psychopathy Measure. A positive association between boldness and inhibition was found, while meanness accounted for the lack of inhibitory control. In addition, disinhibition explained updating dysfunction. These findings provide empirical evidence for dissociable effects of psychopathic traits on executive functioning, in light of the Triarchic Model of Psychopathy.

## Introduction

The evolutionary development of the prefrontal cortex and the expression of the executive functioning (EF) is a distinctive aspect of the human species that originated unprecedented adaptation capabilities (Miyake and Friedman, 2012). EF is defined as a set ofcognitive abilities that promote the successful engagement in independent, goal-oriented, and self-serving behavior (Lezak et al., 2004). However, rather than a unitary construct, EF is an umbrella for several cognitive processes and subprocesses (Elliott, 2003).

The Model of Unity and Diversity (for a review see Miyake et al., 2000) was formulated to systematize the main components of EF, based on the assumption that selective deficits should not determine a general executive dysfunction. Three executive processes are proposed: *shifting* (the ability to shift back and forth between multiple mental sets, requiring the performance of a new operation in face of proactive interference, such as task rules constantly changing); *updating* (active encoding and manipulation of relevant information in the working memory); and *inhibition* (inhibition of automatic and dominant responses).

Basic and complex adaptive behaviors, such as inhibition and decision-making, are based on interactions between the above executive components (Miyake et al., 2000; Miller and Cohen, 2001). In turn, deficits in EF are consistently implicated in antisocial behavior (Morgan and Lilienfeld, 2000; Ogilvie et al., 2011). A robust association (*d* = 0.62–1.09) between antisocial behavior and executive dysfunction is well documented in the literature (Morgan and Lilienfeld, 2000; Ogilvie et al., 2011). However, the link between psychopathy and EF is weak (*d* = 0.29–0.42). This result seems to reflect inconsistent findings in the literature, as some studies found worse performance on EF tasks in psychopaths (e.g., Dolan and Anderson, 2002), while others did not find evidence for executive deficits (e.g., Dvorak-Bertsch et al., 2007).

Conceptual and methodological shortcomings may help to explain conflicting findings. First, a worse performance may be allocated to specific executive components, rather than a general EF deficit (Ogilvie et al., 2011; Bagshaw et al., 2014; Baskin-Sommers et al., 2015). Second, the association between psychopathy and antisocial behavior is not linear and is still under debate. Some authors conceptualize psychopathy as a criminogenic personality structure (Wilson and Herrnstein, 1985), explaining recidivism and violent offenses (Hemphill et al., 1998), but others argue that antisocial behavior features in psychopathy may be a secondary outcome of the core-personality features of psychopathy that are moderated by protective and risk factors (e.g., Cooke and Michie, 2001; Gao and Raine, 2010). In this sense, antisocial behavior may co-occur, or not, with psychopathic personality core-features (Cooke and Michie, 2001; Gao and Raine, 2010), such as shallow affect, superficial charm, manipulativeness, lack of empathy (interpersonal-affective features; Hare, 1991). Executive dysfunction may constitute one of the risk factors for getting apprehended (Ishikawa et al., 2001), while intact EF may be a protective factor associated with the positive adjustment traits of psychopathy (Patrick et al., 2009). In fact, Cleckley (1976) observed the occurrence of this personality structure in subclinical groups. The so-called successful psychopaths are capable of engaging in social rules with no apparent criminal record (Glenn et al., 2011). Finally, the use of cut-off scores to analyze psychopathy as a homogeneous and taxonomic group may be masking the differential associations between specific psychopathic traits and executive deficits (Ogilvie et al., 2011).

From the outlined limitations, dimensional models of psychopathy may be an informative venue to unveil the main differential associations, as proposed here and recently evidenced in a systematic review conducted by Maes and Brazil (2013). Regarding the inhibition component of EF, three studies found better executive performance in adaptive psychopathic traits indexing fearlessness features, such as social efficacy and stress immunity (Sadeh and Verona, 2008; Carlson and Thái, 2010; Feilhauer et al., 2012). In turn, two studies reported impaired inhibition in impulsive–antisocial dimensions that are associated with disruptive and maladaptive behavior (Sellbom and Verona, 2007; Feilhauer et al., 2012). For shifting, the disposition toward low fear resulted on improved performance (Sellbom and Verona, 2007), although the affective dimension (i.e., coldheartedness, meanness, callous-unemotional traits) of psychopathy was related with worse shifting performance (Mahmut et al., 2008). Accordingly, a positive association was observed between better performance on updating tasks and higher scores on fearlessness dimensions of psychopathy (Hansen et al., 2007; Sellbom and Verona, 2007). Altogether, previous findings provide some support for a link between adaptive psychopathic personality traits indexing fearlessness features and better EF, whereas antisocial-impulsive dimensions of psychopathy seem to be associated with impaired inhibitory control.

Despite the above-mentioned findings, Maes and Brazil (2013) called for more empirical research to strengthen the evidence on the connections between specific psychopathy phenotypes and EF components. Specifically, Baskin-Sommers et al. (2015) acknowledged that measures designed from normal personality models may capture the adaptive features of psychopathy to a greater extent than the ones designed from clinical observations. In this sense, the Triarchic Model of Psychopathy (Patrick et al., 2009) may add value to the existing body of literature. This model was designed to integrate the distinct conceptualizations of psychopathy (Patrick et al., 2009), while capturing the positive core-features that were described by Cleckley (1976), though excluded from the criminal conceptualizations. Three psychopathic phenotypes are presented in the model: (a) *meanness*, which comprises lack of empathy, callousness, emotional detachment, active exploitativeness, excitement seeking, rebelliousness, instrumental or predatory aggression, abuse of others, and empowerment through cruelty; (b) *disinhibition*, characterized by a propensity toward problems of impulse control, deregulated negative affect, deficits in foresight, impatient urgency, non-planfulness, low frustration tolerance, reactive aggression, irresponsibility, and vulnerability to substance abuse; and, (c) *boldness*, characterized by optimism, resilience to stress, courage, social dominance, persuasiveness, tolerance for uncertainty, self-confidence, social assurance, and intrepidness (Patrick et al., 2009; Venables et al., 2014; Patrick, 2010, Unpublished). The Triarchic Model asserts that the main psychopathic components are distributed in a continuum, allowing to measure the distinct expressions of psychopathy in community samples, while accounting for both affective-interpersonal and behavioral-impulsive expressions of psychopathy (Patrick et al., 2009). These heterogeneous manifestations of psychopathy are explained by distinct etiological pathways: *externalizing vulnerability* underlies meanness and disinhibition phenotypes, while *low fear* leads to meanness and boldness (Patrick and Bernat, 2009).

The dissociation of etiological paths highlights the importance of refining the psychobiological and behavioral correlates of each phenotype and brings some implications to assess executive deficits in psychopathy. Importantly, extreme expressions of meanness and disinhibition are systematically included in the prototypical conceptualizations of criminal psychopathy (Patrick and Drislane, 2015). Disinhibition reflects an externalizing component of psychopathy, related to deviant behaviors in child and adult populations (Patrick et al., 2009). Empirical data supports the link between externalizing vulnerability and antisocial behavior (Patrick et al., 2005; Gao and Raine, 2010; Kennealy et al., 2010), and it was recently argued that executive dysfunction is the main path to explain antisocial behavior (Morgan and Lilienfeld, 2000; Ogilvie et al., 2011; De Brito et al., 2013). Findings suggest that antisocial individuals show executive impairments regardless of psychopathic features (De Brito et al., 2013). In light of this, disinhibition is the main predictor of impulsive-reactive forms of aggression, and may reflect abnormal prefrontal functioning, which is associated with impaired EF (Patrick et al., 2009). Krueger et al. (2002) pointed that the high comorbidity between different manifestations of externalizing disorders (e.g., Conduct Disorder, Antisocial Personality Disorder, and Substance Dependence) implies shared causal processes. Executive dysfunction may constitute, precisely, one point of intersection over the Externalizing Spectrum. On the contrary, boldness may explain adaptive processes (Patrick et al., 2009) and may be dissociated from executive deficits. In particular, boldness is considered a critical trait to differentiate the positive adjustment features of psychopathy from antisocial behavior (Wall et al., 2015). Boldness seems to constitute an adaptive feature on daily life, given the ability of individuals who score high on boldness to remain calm and concentrated in stressful situations, and to quickly recover from such events (Patrick et al., 2005, 2009; Kennealy et al., 2010; Stanley et al., 2013; Venables et al., 2014; Patrick and Drislane, 2015; Pasion et al., 2016, 2017). Moreover, boldness entails high self-assurance and social efficacy, tolerance for unfamiliarity and danger, social dominance, thrill seeking without anticipatory fear, and emotional resiliency.

Despite the accumulated data, to our knowledge the Triarchic Model of Psychopathy remains untested in studies assessing EF. Recently, Patrick and Drislane (2015) argued that a comprehensive validation of the Triarchic Model demands for linkages between psychopathic phenotypes and measures of brain and behavior. The current study aims to provide direct evidence for the dissociable effects of the psychopathy phenotypes, as proposed by the Triarchic Model, while predicting inhibition, shifting and updating components. Dissociable effects on EF among the phenotypic expressions of psychopathy would allow to validate the triarchic model as a promising venue to explore and refine the neurobehavioral correlates of specific psychopathic manifestations. The use of an assessment model based on normal-range continua of personality traits will further allow to test if executive deficits may be detected in low and moderate levels of the spectra, namely in subclinical samples (Maes and Brazil, 2013).

Considering the externalizing-antisocial behavior link, it is hypothesized that externalizing vulnerability (meanness and disinhibition phenotypes) accounts for an executive dysfunction characterized by low inhibition. Boldness, as a positive adjustment phenotype, is expected to be associated with intact or even improved EF in all its components.

## Materials and Methods

### Sample

This study included 104 male participants, aged between 18 and 60, recruited from forensic (*n* = 56, four left-handed) and community settings (*n* = 48, three left-handed). **Table 1** summarizes sociodemographic data.

**Table 1:** Means (standard deviations) of socio-demographic variables, TriPM scores, and executive performance for the non-forensic and forensic groups.

	Non-forensic (*n* = 48)	Forensic (*n* = 56)
**Socio-demographic variables**		
Age	32.0 (11.6)	39.0 (9.97)
Years of education	13.7 (4.51)	7.71 (2.90)
Past substance abuse (%)	0.00%	49.0%
Recidivism (%)	–	28.6%
**TriPM score**		
TriPM total score	57.4 (19.7)	60.8 (19.7)
Disinhibition	15.3 (7.9)	21.4 (10.1)
Meanness	12.8 (8.2)	12.0 (6.6)
Boldness	29.3 (8.7)	25.3 (8.1)
**EF**		
Inhibition	4.47 (9.48)	1.61 (7.54)
Shifting	45.4 (34.2)	73.9 (64.9)
Updating	3.03 (1.31)	2.24 (1.48)

The forensic group comprised individuals currently convicted for one or more crimes. The recidivism rate was 28.6% and the criminal charges represented the expected criminal versatility (kidnapping, homicide, domestic violence, fraud, theft, and drug dealing) (Benson and Moore, 1992; Piquero et al., 2007; Piquero, 2008; Gavin and Hockey, 2010). The community group (non-forensic) did not report current or previous criminal activities.

Participants were excluded based on the following criteria: foreign nationality, illiteracy, age above 60, diagnosis of psychopathology, neuropathology, cognitive impairment, sensory or motor deficits. Criminal records were reviewed for this purpose and a semi-structured interview was designed to collect more information on exclusion criteria. Cognitive impairment was further screened using the Montreal Cognitive Assessment (MoCA; Nasreddine et al., 2005, adapted by Simões et al., 2008).

### Materials and Stimuli

#### Psychopathy Measures

The TriPM (Patrick, 2010, Unpublished; adapted by Vieira et al., 2014) is a self-report scale with 58 items measuring three psychopathy phenotypes: Boldness, Meanness, and Disinhibition. Responses are provided on a 4-point Likert scale (0 – *false;* 1 – *somewhat false;* 2 – *somewhat true;* 3 – *true*). The boldness subscale (α = 0.853) indexes adaptive features of psychopathy, such as low anxiousness, venturesomeness, and social dominance. The disinhibition subscale (α = 0.844) entails the purest externalizing factors, namely hostility, irresponsibility, and impulsiveness. The meanness subscale (α = 0.868) comprises lack of empathy and close attachment, rebelliousness, excitement seeking, callousness and cruelty. Meanness correlated moderately with disinhibition (*r* = 0.619, *p* < 0.001) and low with boldness (*r* = 0.291, *p* = 0.003). A non-significant correlation was found between boldness and disinhibition (*r* = 0.148, *p* = 0.135), providing support for the etiological differentiation underlying the distinct phenotypic expressions of psychopathy (**Supplementary Tables S1**, **S2** present the associations between phenotypic components for each group). The total score on psychopathy was obtained from the sum of the three subscales. All the fields were required to complete and there were no missing values.

#### Executive Functioning Measures

##### Inhibition

In variants of the original Stroop Color-Word Test it is suggested that 55% of the variance of the task performance is explained by a factor related with the suppression of automatic responses (Miyake et al., 2000; Friedman and Miyake, 2004; Hull et al., 2008). The Stroop (paper-and-pencil version; Stroop, 1935; Portuguese version by Fernandes, 2013) presents a word condition (W; where the participant is asked to read words – red, green and blue – printed in black), a color condition (C; where the participant is asked to name the colors of ‘Xs’ printed in blue, red, and green), and a color/word condition (CW; where the participants should name colors that do not match with the written word). Each condition contains 100 stimuli, horizontally distributed in five columns, to be read during 45 s. The interference ratio (Fernandes, 2013), a widely used measure that removes the confounding factor of processing speed when assessing inhibition, was calculated by the following formula:

$$\text{C}{\text{W}}^{\prime }=\frac{\text{W}\times \text{C}}{\text{W}+\text{C}}$$

##### Updating

Updating explains in 46% the performance variance during the N-Back (Friedman et al., 2008). For this reason, a computer-based spatial 2-Back task (adapted from Kirchner, 1958) was used to assess updating. In the 2-Back task, participants were asked to signal whenever a white square (1000 ms, 2.5 seg of inter-stimuli interval) in a matrix of 3^∗^3 squares was displayed in the same position as two trials before (25 targets, and 71 non-targets). Four measures were obtained from this task: hits (signal is present), misses (signal is present, but the participant do not signal it), false alarms (participant indicates that signal is present when it is not), and correct omissions (participant correctly indicates that there is no signal, by not responding). Sensitivity to the signal was calculated from this task as:

$$\begin{array}{l} d^{\prime }=\Phi^{-1}\left(\frac{\text{hits}}{\left(\text{hits}+\text{misses}\right)}\right)\\ \;-\Phi^{-1}\left(\frac{\mathrm{false alarms}}{\left(\mathrm{false alarms}+\mathrm{correct omissions}\right)}\right) \end{array}$$

The Φ^−1^ function returns the inverse of the standard normal cumulative distribution assuming a distribution with average 0 and sigma 1 (NORMSINV Excel); that is, it transforms Hit Rate (signal is present) and False Alarm Rate (signal is absent) into *z*-scores. Perfect scores were adjusted by the following formulas (Macmillan and Creelman, 1991), where “*n*” refers to the number of hits or false alarms:

$$\begin{array}{c} \mathrm{Hits}=\frac{1}{\left(2n_{\mathrm{hits}}\right)}\\ \mathrm{False Alarms}=\frac{1}{\left(2n_{\mathrm{false alarms}}\right)} \end{array}$$

Two participants from the non-forensic group and three from the forensic group gave up the task, so their results were not considered.

##### Shifting

The paper-and-pencil version of the Trail Making Test (TMT; Reitan and Wolfson, 1985) was administered, considering that t the participants to connect, in an ascending order, a sequence of numbers in circles (part A – from 1 to 24), and a sequence of numbers and letters alternately (Part B – from A to L and 1 to 13). Errors were immediately corrected in both conditions. The task was discontinued after 200 s in Part A, and 400 s in Part B, or after four accumulated errors in each part, except when only three or less circles were lacking to the end. This rule led to the exclusion of 11 participants of the forensic group, and three non-forensic participants. The shifting measure was calculated by subtracting the execution time of Part A from Part B.

### Procedure

The forensic group was recruited from three maximum security prisons. Full access to criminal records was given to assess exclusion criteria. Offenders were selected based on the available information from the files and in collaboration with case workers.

The non-forensic group was recruited by e-mail advertisement using the mailing list of the university campus. In this advertisement, participants were required to complete an on-line version of the TriPM (*n* = 1072). Individuals for the non-forensic group were then selected according to their TriPM scores in order to match the psychopathy scores of the forensic group. A total of 86 participants were invited for the neuropsychological assessment (acceptance rate ( 55.8%).

Individual interviews were conducted to complete demographic data and further screen for exclusion criteria. The neuropsychological tasks were individually administered by two trained psychologists and the order of these tasks was randomized across participants.

Informed consent was obtained from all participants. Ethical principles and code of conduct were strictly followed.

## Results

### Preliminary Analysis

There were significant differences between groups regarding age, *t*(102) = 3.32, *p* = 0.001, and years of formal education, *t*(102) = 8.25, *p* < 0.001 (**Table 1**). None of the participants reported substance use at the time of the study. However, 27 inmates reported past substance abuse.

The groups were matched for the total psychopathy score (*t* < 1), to ensure that significant differences in phenotypic components were not explained by the variation in the total psychopathy score. The total scores ranged between 26 to 102 in the forensic group, and 30 to 116 in the non-forensic group.

Despite similar values in total psychopathy score, group differences (independent variable) emerged when analyzing phenotypic components in a multivariate model (MANOVA) with Bonferroni correction, *T*^2^ = 0.324, *F*(3,100) = 10.8, *p* < 0.001, $\eta_{\text{p}}^{2}$ = 0.245. *Boldness* scores were higher in the non-forensic group, *F*(1,102) = 5.74, *p* = 0.018, $\eta_{\text{p}}^{2}$ = 0.053, while *disinhibition* scores were higher in the forensic group, *F*(1,102) = 11.4, *p* = 0.001, $\eta_{\text{p}}^{2}$ = 0.100, *p* = 0.001 (**Table 1**). The differences in groups regarding recidivism (independent variable), *T*^2^ = 0.324, *F*(3,39) = 4.44, *p* = 0.009, $\eta_{\text{p}}^{2}$ = 0.254, showed that *meanness*, *F*(1,41) = 13.94, *p* = 0.001, $\eta_{\text{p}}^{2}$ = 0.254, and *disinhibition*, *F*(1,41) = 5.07, *p* = 0.030, $\eta_{\text{p}}^{2}$ = 0.110, were higher in the recidivist group, compared to the non- recidivist group. The group reporting past substance use (independent variable), *T*^2^ = 0.324, *F*(3,93) = 8.74, *p* < 0.001, $\eta_{\text{p}}^{2}$ = 0.220, showed higher level of *disinhibition*, *F*(1,95) = 22.7, *p* < 0.001, $\eta_{\text{p}}^{2}$ = 0.193.

Regarding executive functions, *updating*, *t*(98) = 2.71, *p* = 0.007, *d* = 0.56, and *shifting* components, *t*(88) = 2.60, *p* = 0.011, *d* = 0.55, were lower in the forensic group, compared to the non-forensic group (cf. **Table 1**). This effect was not confirmed for *inhibition*, *t*(102) = 1.72, *p* = 0.089, *d* = 0.33.

### EF and Psychopathic Traits

Hierarchical Linear Regression models were used to examine the variance of the executive components explained by psychopathy phenotypes. The models were run independently, considering Miyake et al. (2000) rationale and the absence of significant zero-order correlations across tasks (**Supplementary Table S3**).

The dissociable effects of phenotypic expressions of psychopathy (*boldness*, *meanness*, and *disinhibition*) in EF components (*inhibition*, *updating*, and *shifting*) were analyzed. Group differences and non-matched variables systematically identified in the literature as predictors of EF, were introduced in the model to control moderation effects, if displaying significant correlations with the executive performance (**Supplementary Table S1**). Age and years of education were entered, respectively, in updating and shifting models to control for moderation effects. No evidence for multicollinearity was found in the regression models.

#### Inhibition

*Meanness*, β = 0.318, *t* = 2.51, *p* = 0.014, was the main predictor of *inhibition*. An acceptable power was achieved (85%), despite the non-significance of the regression model, *F*(3,103) = 2.51, Adj *R*^2^ = 0.042, *p* = 0.063, and a small effect size (*R*^2^ = 0.070). The inclusion of the group moderation effects in the model lead to a non-significant increase in *R*^2^ of 0.023 (*p* = 0.118), but the moderation model reached significance, *F*(4,103) = 2.53, Adj *R*^2^ = 0.056, *p* = 0.045. The power increased to 94%. Nevertheless, group was a non-significant predictor of *inhibition*, β = 0.174, *t* = 1.58, *p* = 0.118. Interestingly, *boldness* emerged as a significant predictor, β = −0.210, *t* = 2.04, *p* = 0.044, in an opposite direction of *meanness*, β = 0.260, *t* = 1.98, *p* = 0.051 (**Table 2**).

**Table 2:** Regression model for inhibition (Stroop task).

Model	*F*	*Adj R*^2^	*p*	*R*^2^	*R*^2^ change	Sig. *R*^2^ change	β	*t*	*p*	Power
**Phenotypes**	2.51	0.042	0.063	0.070						0.850
Meanness							0.318	2.51	0.014	
										
Boldness							−0.173	1.72	0.089	
Disinhibition							−0.187	1.52	0.132	
**Moderators**	2.53	0.056	0.045	0.093	0.023	0.118				0.926
Group							0.174	1.58	0.118	
Meanness							0.260	1.98	0.051	
Boldness							−0.210	2.04	0.044	
Disinhibition							−0.090	0.658	0.512	

#### Updating

*Disinhibition* was the main predictor of *updating, F*(3,98) = 3.66, Adj *R*^2^ = 0.075, *p* = 0.015, β = −0.321, *t* = 2.59, *p* = 0.011. In boldness there was a trend toward an opposite pattern, although the effect did not achieve statistical significance, β = 0.198, *t* = 1.95, *p* = 0.055. The moderation effect of *age*, β = −0.271, *t* = 2.42, *p* = 0.017, and *group*, β = 0.069, *t* = 0.606, *p* = 0.546, *F*(5,98) = 3.90, Adj *R*^2^ = 0.129, *p* = 0.003, increased significantly the *R*^2^, *p* = 0.023, to a medium effect size (*R*^2^ = 0.173). *Disinhibition* remained as a significant predictor of updating, β = −0.272, *t* = 2.01, *p* = 0.047, but boldness did not achieve significance in the moderation model, β = 0.129, *t* = 1.26, *p* = 0.211. The observable power was high in both models (94 and 99%, respectively) (**Table 3**).

**Table 3:** Regression model for updating (N-back).

Model	*F*	*Adj R*^2^	*p*	*R*^2^	*R*^2^ change	Sig. *R*^2^ change	β	*t*	*p*	Power
**Phenotypes**	3.66	0.075	0.015	0.104						0.938
Meanness							0.152	1.19	0.238	
Boldness							0.198	1.95	0.055	
Disinhibition							−0.321	2.59	0.011	
**Moderators**	3.90	0.129	0.003	0.173	0.070	0.023				0.993
Group							0.069	0.606	0.546	
Age							−0.271	2.42	0.017	
Meanness							0.023	0.171	0.864	
Boldness							0.129	1.26	0.211	
Disinhibition							−0.272	2.01	0.047	

#### Shifting

The model including the phenotypic expressions of psychopathy did not reach significance and did not yielded significant predictions on *shifting* (**Table 4**). The *post hoc* power was low (31%). The moderation analysis lead to a significant *R*^2^ increase of 0.120, *p* = 0.004, and on power (97%). *Years of education* was the main moderator accounting for the significance and medium effect size (*R*^2^ = 0.136) of the model*, F*(5,89) = 2.64, Adj *R*^2^ = 0.084, *p* = 0.029, and explained improved *shifting*, β = −0.334, *t* = 2.46, *p* = 0.016 (**Table 4**).

**Table 4:** Regression model for shifting (TMT).

Model	*F*	*Adj R*^2^	*p*	*R*^2^	*R*^2^ change	Sig. *R*^2^ change	β	*t*	*p*	Power
**Phenotypes**	<1	−0.019	0.722	0.015						0.311
Meanness							−0.110	0.778	0.439	
Boldness							−0.012	0.109	0.913	
Disinhibition							0.154	1.13	0.261	
**Moderators**	2.64	0.084	0.029	0.136	0.120	0.004				0.966
Group							−0.109	0.803	0.424	
Education							−0.344	2.47	0.016	
Meanness							0.035	0.247	0.805	
Boldness							0.127	1.12	0.267	
Disinhibition							−0.088	0.588	0.558	

## Discussion

The link between antisocial behavior and executive dysfunction is well-established (Morgan and Lilienfeld, 2000; Ogilvie et al., 2011), but when analyses are redirected to psychopathy, empirical findings become less robust (Ogilvie et al., 2011). This result may seem paradoxical, considering the close association between psychopathy and antisocial behavior systematically described in the literature. The current study aimed to clarify the inconsistent findings by dissociating psychopathy phenotypes and executive components. The analysis of specific profiles of psychopathy and separate components of executive functioning may allow a more precise evaluation of its complex relationships.

In a global analysis, our results are consistent with previous findings. A poor performance in updating and shifting was observed in the forensic group, with medium effect sizes. In inhibition the effects were almost significant and small in magnitude, probably due to the small sample size. Thus, differences were found between phenotypic components, confirming that the psychopathic personality structure has heterogeneous features and demands for a dimensional approach. Higher traits of disinhibition were observed in the forensic group, while the non-forensic group exhibited higher traits of boldness. Furthermore, meanness and disinhibition explained recidivism and disinhibition accounted for past substance abuse. Such findings reinforce the thesis that the etiological pathway of externalizing vulnerability is linked to disruptive and antisocial behavior, while boldness remains unrelated to these phenomena (Patrick et al., 2009). This is consistent with the assumption that psychopathy *per se* may not be a risk factor for criminal behavior (Ishikawa et al., 2001). This risk may be moderated by adaptive psychopathy traits, and mediated by other risk factors, such as impaired EF (Cooke and Michie, 2001; Ishikawa et al., 2001; Patrick et al., 2009).

The maladaptive features of psychopathy, mainly related to disinhibition and meanness (externalizing vulnerability), were expected to be associated with the lack of inhibitory control. In turn, we hypothesized that boldness, as an adaptive phenotype, would be associated with intact or even improved EF in all its components.

Our results revealed that the psychopathic traits were the main predictors regarding the inhibition component of EF. Group was a non-significant predictor of inhibition and the increase in *R*^2^ was non-significant and small in magnitude, but moderated a significant dissociation between boldness and meanness traits. Providing support for our hypothesis, boldness was associated with an enhanced ability to inhibit automatic responses, which is in line with previous studies (Sadeh and Verona, 2008; Carlson and Thái, 2010; Feilhauer et al., 2012) and the adaptive role of boldness (Patrick et al., 2009). Conversely, meanness was related with high interference scores, suggesting this phenotype predicts action toward immediate and impulsive behavior. This result is aligned with previous findings on impulsive-antisocial dimensions of psychopathy (Sellbom and Verona, 2007; Feilhauer et al., 2012). The inhibitory deficit in meanness may also help to explain the previously reported association between the affective facet of psychopathy and aggressive-violent behavior (Baskin-Sommers et al., 2015). Meanness might be conceived as a maladaptive phenotypic expression of a fearless temperament associated with life-course antisocial trajectories (Polaschek and Daly, 2013), characterized by greater impulsiveness and aggressiveness. Unexpectedly, higher traits of disinhibition did not predict lower inhibitory control. The lack of a significant association between disinhibition and inhibition does not seem to be explained by inadequate statistical power, which was high in both models (85–93%). Considering that differences on Stroop interference between forensic and non-forensic participants were not significant, it would be necessary to use other tasks to better detect differences in this executive function and test if disinhibition is the best phenotypic candidate to capture poor inhibitory control.

Interestingly, disinhibition predicted reduced sensitivity to the signal during the N-Back. It suggests that updating abilities may play an important role in the executive deficits of individuals characterized by high traits of disinhibition. Updating requires the capacity to dynamically monitor, control, replace, and manipulate information in working memory. If the updating component is affected in disinhibition, the ability to acquire new and relevant information may be compromised, limiting learning from past experiences. To date, studies had only evidenced a positive association between fearlessness-related traits of psychopathy and updating (Hansen et al., 2007; Sellbom and Verona, 2007). Our study adds evidence on the opposite pattern for the disinhibition phenotype. Previously studies on P3 – a neurophysiological correlate of updating – reported P3 blunted amplitude in individuals scoring high on impulsive and antisocial traits of psychopathy (Carlson et al., 2009; Carlson and Thái, 2010; Pasion et al., 2017). Our results seem to be capturing this effect at a behavioral level. In line with the abovementioned studies (Hansen et al., 2007; Sellbom and Verona, 2007), boldness emerged as a predictor of updating. For instance, P3 amplitude is also found to be increased in fearlessness-related traits, as boldness (Pasion et al., 2017). The trend for a dissociable effect with disinhibition strengths the assumption that boldness is an adaptive phenotype, by explaining improved ability to encode and manipulate relevant information in the working memory. Nevertheless, we did not confirm a significant effect of boldness when the moderators were entered in the model and this was not explained by insufficient observed power (99%). A significant negative association was evidenced between age and updating and an increase to a medium effect size was found in the model in which disinhibition, but not boldness, remained a significant predictor of impaired updating. Group was a non-significant moderator.

The performance on shifting was not explained by the distinct phenotypic expressions of psychopathy. This model did not yield significant predictions, the effects were negligible and the achieved power was poor (31%). In turn, the model accounting for moderators explained a significant increase in power to 97% and in *R*^2^ to a medium effect size. Group was a non-significant moderator and years of education emerged as the unique predictor of enhanced shifting abilities. The lack of consistent and significant associations between psychopathic traits and shifting was reported in previous studies (Sellbom and Verona, 2007; Mahmut et al., 2008; Racer et al., 2011; Dolan, 2012), indicating that shifting is not a core-deficit when explaining risk factors for antisocial behavior.

Taken together, the current study highlights that psychopathic personality traits explain in a great extent EF than incarceration. The documented group differences in EF were suppressed when psychopathic traits were introduced in the regression models. Moreover, our study found evidence for the etiological dissociation proposed by Patrick et al. (2009). Meanness and disinhibition were associated with maladaptive behavior (recidivism and past substance abuse), and traits of the disinhibition phenotype were higher in the forensic group. Disinhibition and meanness were found to be moderately correlated and are systematically referred to in the prototypical conceptualization of criminal psychopaths (Patrick et al., 2009). The externalizing manifestation of psychopathy (high disinhibition in combination with high meanness) also evidenced a poor EF. Meanness and disinhibition predicted negatively the performance on inhibition and updating tasks, respectively. Deficits in EF may explain the higher risk for persistent rule breaking as frequently observed in antisocial behavior (Baskin-Sommers et al., 2015). Executive impairments (Ishikawa et al., 2001), as well as reductions of prefrontal gray matter (Yang et al., 2005), were previously observed in criminals showing high scores in the impulsive-antisocial factor of psychopathy.

In turn, a disposition toward low fear may constitute a key component for the adaptive social functioning. The boldness-fearlessness expression of psychopathy (i.e., high boldness scores, in combination with low to moderate meanness) seems to be demarked from criminal correlates and executive impairment. Accordingly, we found higher boldness in the community sample, and these traits accounted for enhanced inhibitory control. Better inhibitory control may prevent disruptive, aggressive and violent behavior to occur in individuals with high boldness traits. The ability to remain calm in stressful and unfamiliar contexts may result in a circumstance that puts these individuals in a favorable position to reach high performance. In a coherent picture, improved EF (Ishikawa et al., 2001), as well as intact gray matter (Yang et al., 2005), were observed in successful (non-criminal) psychopaths that scored lower in impulsive-antisocial factor (Ishikawa et al., 2001) and higher in the interpersonal-affective factor (DeMatteo et al., 2006).

It is important to acknowledge that meanness shares the etiological pathway of low fear with boldness. Nevertheless, the operationalization of meanness in the triarchic psychopathy measure addresses secondary features of externalization designed from the same self-report that operationalizes disinhibition (Patrick, 2010, Unpublished). The behavioral manifestations of meanness comprise arrogance, defiance of authority, a lack of emotional attachment, aggressive competitiveness, physical cruelty and exploitation toward others, premeditated aggression, and excitement seeking through destructiveness (Patrick et al., 2009). Therefore, the link between meanness and externalizing vulnerability seems to be stronger than the one with low fear. Supporting that meanness and disinhibition are close phenotypes, a weak correlation between boldness and meanness was found in our study, in contrast with the moderate association between disinhibition and meanness.

The dissociation of psychopathy traits highlights that dimensional models are promising to clarify conflicting results from studies assessing EF as a single construct and psychopathy as a homogeneous construct. Studies examining EF deficits in psychopathy remained focused on the taxonomic differences between psychopaths and non-psychopaths. Of 191 studies investigating the EF components of the Miyake et al. (2000) model in psychopathic personality only 11 analyzed the distinctive traits of psychopathy (Maes and Brazil, 2013). Moving toward dimensional models will allow to analyze psychopathy in terms of its distinct phenotypes. This may help to shed light on differential relations with brain and behavior that are not evidenced when using total scores.

Although common to most studies in this field, the main methodological limitations of this study should be outlined. Firstly, psychopathy scores were based on self-report measures, which raise questions of social desirability bias, particularly in individuals who may be highly manipulative. Secondly, as we did not cross information with judicial authorities, eventual criminal acts in the community sample may have gone unnoticed. Third, the samples were matched according to the total psychopathy scores. While this procedure allows one to explore the distinct prevalence of phenotypic expressions of psychopathy among samples that do not differ in their variation in terms of total scores, it may also force the selection of non-forensic participants who have high psychopathy scores, and who score highly on certain phenotypes that are not typical of non-forensic participants more generally. Finally, the cross-sectional nature of this study limits the inference of causal relationships between psychopathy phenotypes and EF. Regardless of the above limitations, our results suggest that inconsistent findings regarding EF on psychopathy may be explained, at least partly, by a variable representation of disinhibition, meanness, and boldness phenotypes across samples. Therefore, disentangling the psychopathic personality structure into its distinctive phenotypes may favor an accurate analysis of their specific correlates.

Future research should extend the main findings and accumulate knowledge to establish a robust association between triarchic dimensions of psychopathy and EF, both in forensic and community samples, controlling not only for the criminal trajectory but also related variables such as socioeconomic status and incarceration effects.

## Ethics Statement

This study was carried out in accordance with the recommendations of Declaration of Helsinki, World Medical Association (WMA), and the European Code of Conduct for Research Integrity, All European Academies (ALLEA). The protocol was approved by the Scientific Board of Psychology Doctoral Program. All subjects gave written informed consent in accordance with the Declaration of Helsinki.

## Author Contributions

RP, AC, and FB conceptualized the study. RP and AC collected the data. RP analyzed the data and prepared the paper. AC and FB reviewed the paper. FB supervised the study.

## Conflict of Interest Statement

The authors declare that the research was conducted in the absence of any commercial or financial relationships that could be construed as a potential conflict of interest.
