# Supplementary material for: Dissociable Effects of Psychopathic Traits on Executive Functioning: Insights From the Triarchic Model
Source: Front Psychol. 2018 Sep 12;9:1713. doi: 10.3389/fpsyg.2018.01713 (PMC6144192; doi:10.3389/fpsyg.2018.01713)
Supplement: Supplementary file 1 [file Table_1.DOCX]

Table 1.

*Correlations between TriPM dimensions and executive functioning in the forensic group*

| . | Stroop_ | | | TMT | Nback | Meanness | Boldness | Disinhibition |
| --- | --- | --- | --- | --- | --- | --- | --- | --- |
| Stroop | |  | - | 0.078 | 0.182 | 0.049 | -0.062 | -0.043 |
| TMT | |  |  | - | -0.286 | 0.074 | 0.185 | -0.010 |
| N-Back | |  |  |  | - | -0.031 | 0.142 | -0.029 |
| Meanness | |  |  |  |  | - | .159 | .702** |
| Boldness | |  |  |  |  |  | - | .308* |
| Disinhibition | |  |  |  |  |  |  | - |

**p* < .05; ***p*< .01

Table 2.

*Correlations between TriPM dimensions and executive functioning in the non-forensic group*

|  | Stroop | TMT | Nback | Meanness | Boldness | Disinhibition |
| --- | --- | --- | --- | --- | --- | --- |
| Stroop | - | 0.095 | 0.162 | 0.218 | -0.233 | 0.141 |
| TMT |  | - | -.434^**^ | 0.013 | -0.207 | 0.099 |
| Nback |  |  | - | 0.020 | 0.124 | -0.265 |
| Meanness |  |  |  | - | .399* | .674** |
| Boldness |  |  |  |  |  | .125 |
| Disinhibition |  |  |  |  |  | - |

**p* < .05; ***p*< .01

Table 3.

*Correlations between tasks and non-matched variables, as well as with TriPM dimensions considering the total sample.*

|  | Stroop | TMT | N-Back | Education | Age | Meanness | Boldness | Disinhibition |
| --- | --- | --- | --- | --- | --- | --- | --- | --- |
| Stroop | - | .101 | .264 | .132 | -.232 | .150 | -.107 | -0.17 |
| TMT |  | - | -.321 | -.478* | -.151 | .020 | -.084 | .120 |
| N-Back |  |  | - | .306 | -.415* | -.006 | .181 | -.198* |

**p* < .05; ***p*< .01
